# Aberrant Expressions and Variant Screening of *SEMA3D* in Indonesian Hirschsprung Patients

**DOI:** 10.3389/fped.2020.00060

**Published:** 2020-03-11

**Authors:** Gunadi, Alvin Santoso Kalim, Nova Yuli Prasetyo Budi, Hamzah Muhammad Hafiq, Annisa Maharani, Maharani Febrianti, Fiko Ryantono, Dicky Yulianda, Kristy Iskandar, Joris A. Veltman

**Affiliations:** ^1^Pediatric Surgery Division, Department of Surgery/Genetics Working Group, Faculty of Medicine, Public Health and Nursing, Universitas Gadjah Mada/Dr. Sardjito Hospital, Yogyakarta, Indonesia; ^2^Department of Child Health/Genetics Working Group, Faculty of Medicine, Public Health and Nursing, Universitas Gadjah Mada/UGM Academic Hospital, Yogyakarta, Indonesia; ^3^Faculty of Medical Sciences, Biosciences Institute, Newcastle University, Newcastle upon Tyne, United Kingdom

**Keywords:** aberrant expression, Hirschsprung disease, Indonesia, *SEMA3D*, rare and common variants

## Abstract

**Background:** The *semaphorin 3D* (*SEMA3D*) gene has been implicated in the pathogenesis of Hirschsprung disease (HSCR), a complex genetic disorder characterized by the loss of ganglion cells in varying lengths of gastrointestinal tract. We wished to investigate the role of *SEMA3D* variants, both rare and common variants, as well as its mRNA expression in Indonesian HSCR patients.

**Methods:** Sanger sequencing was performed in 54 HSCR patients to find a pathogenic variant in *SEMA3D*. Next, we determined *SEMA3D* expression in 18 HSCR patients and 13 anorectal malformation colons as controls by quantitative real-time polymerase chain reaction (qPCR).

**Results:** No rare variant was found in the S*EMA3D* gene, except one common variant in exon 17, p.Lys701Gln (rs7800072). The risk allele (C) frequency at rs7800072 among HSCR patients (23%) was similar to those reported for the 1,000 Genomes (27%) and ExAC (28%) East Asian ancestry controls (*p* = 0.49 and 0.41, respectively). A significant difference in *SEMA3D* expression was observed between groups (*p* = 0.04). Furthermore, qPCR revealed that *SEMA3D* expression was strongly up-regulated (5.5-fold) in the ganglionic colon of HSCR patients compared to control colon (ΔC_T_ 10.8 ± 2.1 vs. 13.3 ± 3.9; *p* = 0.025).

**Conclusions:** We report the first study of aberrant *SEMA3D* expressions in HSCR patients and suggest further understanding into the contribution of aberrant *SEMA3D* expression in the development of HSCR. In addition, this study is the first comprehensive analysis of *SEMA3D* variants in the Asian ancestry.

## Introduction

Hirschsprung disease (HSCR: MIM# 142623) is a complex genetic disorder, characterized by the lack of ganglion cells in the bowel, resulting in a functional obstruction during infancy (1). HSCR is categorized into the following types: short-segment, long-segment, and total colonic aganglionosis (1, 2).

HSCR incidence differs among ethnic groups, with 1.5, 2.1, and 2.8 cases per 10,000 live births in European, African, and Asian ancestry cases, respectively (1, 2). There are at least 17 genes responsible for the development of HSCR, with most of them being members of the *RET* and *EDNRB* signaling pathways (1, 2). Two genetic risk factors are the *RET* rs2435357 and rs2506030 variants (3, 4). Our recent studies showed that the *RET* rs2435357 and rs2506030 risk alleles have higher frequency in Indonesian ancestry cases as compared with European ancestry cases (5, 6), which might relate to the higher incidence of HSCR in Indonesia (3.1 cases per 10,000 live births) than other populations (7).

The third signaling pathway of HSCR pathogenesis includes class 3 semaphorins (SEMA3s), involving *SEMA3D* (4, 8, 9) *SEMA3D* has been implicated in the development of HSCR and contributes to risk through both common and rare variants in European ancestries (4, 8, 9), as evidenced by (1) the detection of both common and rare *SEMA3D* variants in HSCR patients; (2) the expression of *SEMA3D* in the human, mouse, and zebrafish intestines and, particularly, the enteric nervous system (ENS); and (3) the joint effect of *Ret* and *Sema3d* loss of function in an aganglionosis animal model. However, our recent study showed that the effect of *SEMA3* rs11766001 common variant on HSCR depends on the ethnic background (10). In addition, the allele frequencies of common variants might differ among Asians, since the North Asians, Han Chinese, Japanese, and Southeast Asians can be distinguished based on their Y chromosome variants (11). Moreover, alterations in the expression of specific genes have been implicated in the development of HSCR (12–15). Therefore, we wished to investigate the role of *SEMA3D* variants, both rare and common variants, as well as its mRNA expression in Indonesian HSCR patients.

## Materials and Methods

### Patients for SEMA3D Variant Screening

We identified 54 HSCR patients: 38 males and 16 females (Table 1). We diagnosed HSCR in these patients in Dr. Sardjito Hospital, Yogyakarta, Indonesia, after evaluating clinical findings, contrast enema, and histopathology. For histopathological findings, we used hematoxylin-eosin staining and S100 immunohistochemistry (5–7, 10, 15, 16).

**Table 1:** Clinical features of the HSCR patients for *SEMA3D* sequencing analysis.

**Clinical features**	***n* (%); months**
**SEX**	
• Male	38 (70)
• Female	16 (30)
**AGANGLIONOSIS TYPES**	
• Short segment • Long segment • Total colon aganglionosis	53 (98) 1 (2) 0
**AGE AT DIAGNOSIS**	34.6 ± 44.5
**AGE AT DEFINITIVE SURGERY**	38.7 ± 43.9
**DEFINITIVE SURGERY (49 PATIENTS)**	
• Transanal endorectal pull-through	21 (43)
• Duhamel	12 (25)
• Transabdominal Soave	11 (22)
• Posterior sagittal neurectomy	4 (8)
• Posterior myectomy	1 (2)

All parents signed a written informed consent form before participating in this study. The Institutional Review Board of the Faculty of Medicine, Public Health, and Nursing, Universitas Gadjah Mada/Dr. Sardjito Hospital gave approval for this study (KE/FK/1356/EC/2015). All experiments were performed in accordance with relevant guidelines and regulations.

### Polymerase Chain Reaction (PCR) and DNA Sequencing

A QIAamp DNA Extraction Kit (QIAGEN, Hilden, Germany) was used to extract genomic DNA from whole blood from each individual, according to the manufacturer's instructions. We stored the extracted DNA samples at −20°C until analysis. PCR was conducted using a Swift Maxi thermal cycler (Esco Micro Pte. Ltd., Singapore), followed by Sanger sequencing analysis to identify sequence variants in all 17 exons of the *SEMA3D* gene in HSCR patients using BigDye Terminator V3.1 Cycle Sequencing Kits (Applied Biosystems, Foster City, CA) and a 3730xl Genetic Analyzer (Applied Biosystems), with DNA Sequencing Analysis Software (Applied Biosystems) 0.1 (7). The primer sequences for *SEMA3D* rare variant analysis were chosen based on a previous study (4).

### DNA Genotyping

DNA genotyping was performed using Sanger sequencing analysis. The *SEMA3D* rs7800072:A>C (chr7: g. 84,628,989A>C) variant was identified during the Sanger sequencing analysis to find a rare variant in Indonesian HSCR patients. The risk allele (C) was determined according to the 1,000 Genomes Project and ExAC population databases (17, 18).

### RNA Extraction and Quantitative Real-Time PCR (qPCR)

The ganglionic and aganglionic intestinal specimens were collected at pull-through surgeries from 18 HSCR patients, while control intestinal specimens were obtained at colostomy closure from 13 anorectal malformation (ARM) patients (Table 2).

**Table 2:** Clinical characteristics of the HSCR patients for *SEMA3D* expression study.

**Clinical characteristics**	***n* (%); months**
**SEX**	
• Male	11 (61)
• Female	7 (39)
**AGANGLIONOSIS TYPES**	
• Short segment • Long segment • Total colon aganglionosis	16 (89) 2 (11) 0
**AGE AT DIAGNOSIS**	16.5 ± 33.3
**AGE AT DEFINITIVE SURGERY**	25.5 ± 35.8
**DEFINITIVE SURGERY**	
• Transanal endorectal pull-through	13 (72.2)
• Duhamel	3 (16.7)
• Transabdominal Soave	2 (11.1)

Total RNA was isolated from colonic specimens using the total RNA Mini Kit (Tissue) (Geneaid Biotech Ltd., New Taipei City, Taiwan). RNA was quantified by a NanoDrop 2000 Spectrophotometer (Thermo Scientific, Wilmington, DE, USA). The OD260/280 ratios ranged from 1.8 to 2.0, indicating high RNA purity.

*SEMA3D* gene expression was quantified using the BioRad CFX Real-Time PCR System (California, USA) and the SensiFAST^TM^ SYBR® No-ROX One-Step Kit (Bioline, Meridian Bioscience, Memphis, USA) using the 5′-CAACGCAGCCTGATAAACAA-3′ (forward) and 5′-TCTTTCATCTCTTGTGGGGAGT-3′ (reverse) (19) The primers were designed to bridge *SEMA3D* exon 8 and 9 junctions (19). *Glyceraldehyde-3-phosphate dehydrogenase* (*GAPDH*), a housekeeping gene, was used as an endogenous control. The *GAPDH* primers were 5′-GCACCGTCAAGGCTGAGAAC-3′ (forward) and 5′-TGGTGAAGACGCCAGTGGA-3′ (reverse). qPCR reactions contained SensiFAST^TM^ SYBR® No-ROX One-Step mix (2×) 10 μL, RiboSafe RNase Inhibitor 0.4 μL, reverse transcriptase 0.2 μL, forward primer (10 μM) 0.8 μL, reverse primer (10 μM) 0.8 μL, and total RNA 50 ng, with final volume of 20 μL. qPCR was performed for 10 minutes (min) at 45°C for reverse transcription process, followed by 2 min at 95°C and 39 cycles for 5 s at 95°C, 10 s at 58°C and, 5 s at 72°C, and 1 cycle for 5 s at 65°C, according to the manufacturer's instructions. We performed the gel electrophoresis for the qPCR of *SEMA3D* and *GAPDH* (Supplemental Figure 1).

The Livak method was utilized to determine the *SEMA3D* mRNA expression level (20). This method is designed to calculate a relative gene expression and referred to as the ΔC_T_ method. The (log) expression is proportional to the negative C_T_ value (the lower the C_T_, the higher the expression) (20).

### Statistical Analysis

*SEMA3D* expression was described as a mean value ± SD. The Kolmogorov–Smirnov test was used to determine the data distribution, and a one-way ANOVA was utilized to assess statistical differences between groups. A chi-square test was used to establish *p*-value for the case-control association analysis for *SEMA3D* rs7800072 variant. A *p* < 0.05 was considered significant.

## Results

Most of our HSCR patients were male (70%) and short-segment aganglionosis type (98%), with the mean age at diagnosis being 34.6 ± 44.5 months (Table 1). Among the 54 HSCR patients, 49, 3, and 2 children underwent a definitive surgery, a colostomy, and a full-thickness rectal biopsy waiting a pull-through procedure, respectively, with the most common definitive surgery conducted being transanal endorectal pull-through (43%) (Table 1); and our controls were six males and seven females, with a mean age during stoma closure of 47 ± 45.1 months.

**Figure 1:**
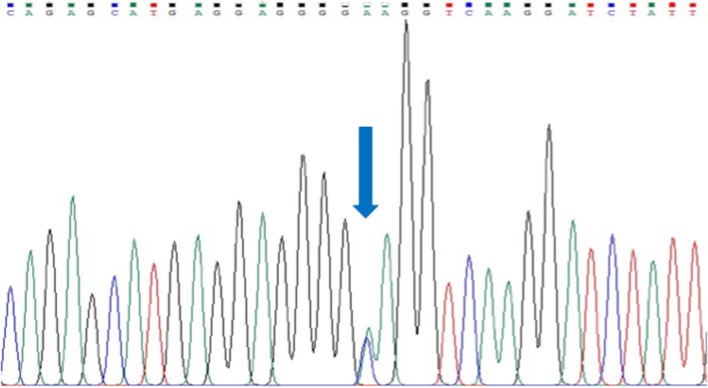
Sanger sequencing of exon 17 *SEMA3D* gene in a HSCR patient. Arrow indicates a common variant, p.Lys701Gln (rs7800072).

We could not identify any rare variant in all 17 exons of *SEMA3D* gene in 54 HSCR patients, but encountered one common variant in exon 17: p.Lys701Gln (rs7800072) (Figure 1). The genotype frequencies for rs7800072 variant among HSCR patients were as follows: AA (32/54, 60%), AC (19/54, 35%), and CC (3/54, 5%). Subsequently, we compared the risk allele (C) frequency of rs7800072 in 54 Indonesian HSCR cases and the 1000 Genomes and ExAC East Asian ancestry controls (Table 3). The risk allele (C) frequency was similar in HSCR cases (23%) and the 1000 Genomes (27%) and ExAC (28%) East Asian ancestry controls, with *p*-values of 0.49 and 0.41, respectively (Table 3). According to the conservation score prediction using PhyloP, the p.Lys701Gln variant did not reach a deleterious threshold of 0.84, while the predicted deleterious effect (Condel) showed the p.Lys701Gln variant as being neutral (4). Furthermore, the SIFT and PolyPhen-2 analysis of p.Lys701Gln showed the variant as being tolerated and benign, respectively (18).

**Table 3:** *SEMA3D* common variant frequency in Indonesian HSCR and population databases (17, 21).

**Variant (risk allele)**	**HSCR patients**	1000 Genomes^*^	ExAC^*^	***p*****-value**	
				**vs. 1000 Genomes**	**vs. ExAC**
p.Lys701Gln (rs7800072) (C)	25/108	274/1,008	2,399/8,626	0.49	0.41

* *East Asian ancestries; ExAC, exome aggregation consortium; HSCR, Hirschsprung disease*.

**Figure 2:**
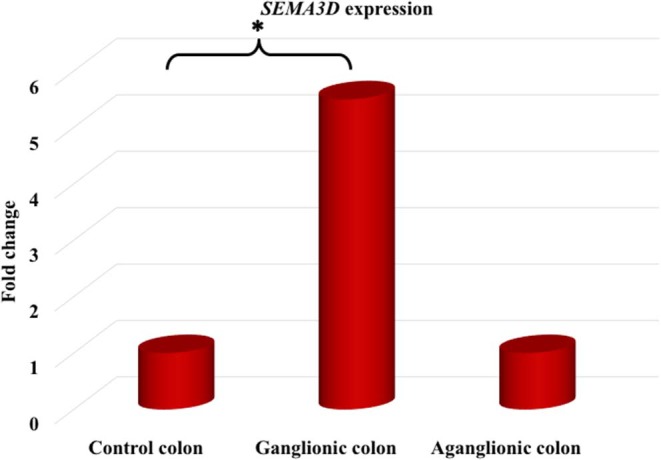
The *SEMA3D* expression was strongly up-regulated (5.5-fold) in the ganglionic colon of HSCR patients compared to control colon (*p* = 0.025), while the *SEMA3D* expression was not significantly different between the aganglionic colon of HSCR patients and the control colon (*p* = 0.89). *p < 0.05.

**Figure 3:**
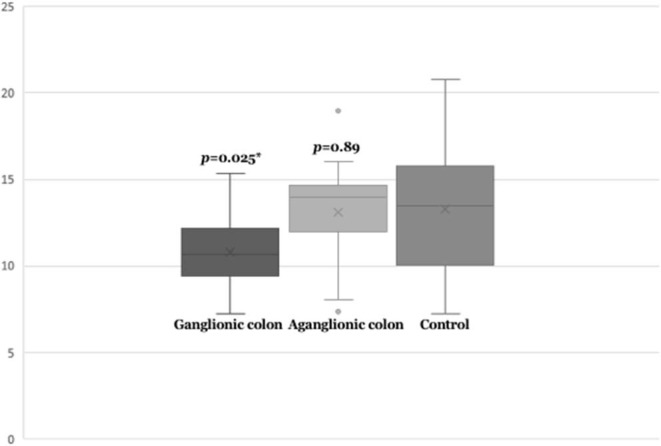
Box-plot graph of ΔC_T_ value of the *SEMA3D* expressions in HSCR ganglionic colon (**ΔC**_**T**_ 10.8 ± 2.1), HSCR aganglionic colon (**ΔC**_**T**_ 13.4 ± 2.9), and control colon (**ΔC**_**T**_ 13.3 ± 3.9). Box-plot graph of ΔC_T_ value reveals the median values as lines across the box. Lower and upper boxes are representing the 25th percentile to the 75th percentile, while whiskers indicate the maximum and minimum values. *p < 0.05.

Next, we compared *SEMA3D* expression in 18 aganglionic and ganglionic colons of HSCR patients and 13 control colons. The distribution of *SEMA3D* expression data were normal for ganglionic, aganglionic, and control colons (*p* = 0.2, 0.17, and 0.2, respectively). A significant different of *SEMA3D* expression was observed between groups (*p* = 0.04). Furthermore, qPCR showed that the *SEMA3D* expression was strongly up-regulated (5.5-fold) (Figure 2) in the ganglionic colon of HSCR patients compared to control colon (**ΔC**_**T**_ 10.8 ± 2.1 vs. 13.3 ± 3.9; *p* = 0.025) (Table 4, Figure 3), while the *SEMA3D* expression was not significantly different between the aganglionic colon of HSCR patients and the control colon (**ΔC**_**T**_ 13.1 ± 3.0 vs. 13.3 ± 3.9; *p* = 0.89) (Table 4, Figure 3).

**Table 4:** *SEMA3D* expressions in colon of HSCR patient and control.

***SEMA3D***	**ΔC_**T**_ ± SD**	**ΔΔC_**T**_ (95% CI)**	**Fold change**	***p*-value**
Ganglionic colon	10.8 ± 2.1	−2.5 (−4.7 to −0.2)	5.5	0.025^*^
Aganglionic colon	13.1 ± 3.0	−0.2 (−2.4 to 2.1)	1.1	0.89
Control colon	13.3 ± 3.9			

*CI, confidence interval; C_T_, cycle threshold; SD, standard deviation*;

* *p < 0.05 is considered statistically significant*.

## Discussion

In this study, we have performed an in-depth genetic and gene expression study of the *SEMA3D* in Indonesian HSCR patients. We did not detect any rare variants in this gene, although previous studies in European ancestry cases have identified rare coding variants in *SEMA3D* associated with HSCR. Our results indicate that the association of such variants to the disease may be restricted to specific ethnic groups. It may still be the case that other *semaphorin 3* genes could play a role in HSCR pathogenesis (4, 9). A previous study showed the existence of other genetic factors conferring risk to HSCR in specific ethnic populations (3). For example, there were two different *RET* haplotypes involving the enhancer mutation that were over-transmitted to the HSCR offspring in the European sample, while in the Chinese sample, only one of those haplotypes was present (3). It might be speculated that the enhancer mutation arose in one haplotype, which after the Asian–Caucasian split, rearranged to also give the other haplotype, but exclusively in the Caucasian (22). Another example is the *NRG1* rs7835688 genetic marker, which has been originally discovered in Chinese HSCR patients (23) and has been shown in other Asian ancestry cases (5, 24), but is rare and shows no effect in the Caucasian population (8, 22). Makhmudi et al. (25) also demonstrated that the *MTHFR* c.677C>T is a genetic risk factor for Indonesian gastroschisis, but not seen in Caucasians (26, 27). A recent study showed that loss of *Sema3d* in null homozygotes mice had no impact on the intestinal transcriptome (28). The authors were unable to find evidence for *Ret* and *Sema3d* interaction affecting survival, presence of myenteric plexus, or intestine transcriptome (28). Furthermore, Tang et al. revealed that the effects of *RET* and *NRG1* variants are universal across Caucasian and Asian ancestries, but the impact of *SEMA3* variant was restricted to Caucasian ancestries (29). It should be noted that our screening method did not cover the promoter and enrich regions of CpG of *SEMA3D*.

In addition, the observed *SEMA3D* rs7800072 variant frequency in Indonesian HSCR patients is similar to those reported for the 1,000 Genomes Project and ExAC East Asian ancestry controls (17, 18). Therefore, we might conclude that this common variant does not have a role on the development of HSCR in Indonesia.

ENS development is a complex process, regulated by a large range of molecules and signaling pathways. This strictly controlled process needs the correct regulation of ENS-specific gene expression. The expressions of several genes implicated in HSCR development have been shown to be regulated by epigenetic mechanisms. *RET* expression was regulated by retinoic acid through DNA methylation on this 5′-CG-3′-rich enhancer region (30), while a significantly lower level of *EDNRB* methylation was also detected in HSCR patients (31). Interestingly, our study clearly demonstrates that *SEMA3D* gene expression was strongly up-regulated in the ganglionic intestines of HSCR patients as compared to controls. To the best of our knowledge, our study is the first report of the aberrant expressions of *SEMA3D* in HSCR patients. Another novelty in our findings is we conducted the study on patients with Indonesian ancestry [vs. European (4) ancestry cases]. It has been shown that reduced *sema3d* expression by morpholino resulted in severe effects on the intestine and its innervation of zebrafish (4). In addition, *sema3d* bound to *nrp1a* to facilitate the axonal guidance and contributed to peripheral axon outgrowth interdependently with *dpysl3* (32, 33) Binding of Sema3d to neuropilin and plexin receptors introduced biochemical responses in specific neurons and stimulated the neuron migration (34, 35). Therefore, we might hypothesize that the aberrant expressions of *SEMA3D* will have an impact in our HSCR patients by affecting the neuronal guidance during ENS development.

It has been shown that some HSCR patients have persistent bowel symptoms, such as constipation, soiling, and enterocolitis, after an appropriate pull-through procedure. Most HSCR patients with persistent bowel symptoms do not have any identifiable etiology for their ongoing bowel dysmotility (36). The current hypothesis is that the aberrant expression of some genes in the ganglionic colon of HSCR patients includes *SK3, Cx26, ChAT*, and *nNOS* (37–40). Our study presented the altered *SEMA3D* expressions in the ganglionic colon of HSCR patients. Therefore, we might hypothesize that the aberrant *SEMA3D* expressions in the ganglionic colon involve in the pathogenesis of persistent bowel symptoms in HSCR patients following a properly performed pull-through surgery.

Our results should be interpreted with some caution, however, because they are based on overall means without accounting for other factors generating variation in the data such as gender, age, and degree of aganglionosis. Further studies of the methylation pattern of the *SEMA3D* gene are required to investigate whether the aberrant expression of *SEMA3D* in HSCR patients is due to abnormal DNA methylation. It is also necessary to compare the *SEMA3D* protein expression level between HSCR patients and controls and to identify its location in the colon tissue to prove the increased *SEMA3D* expressions. Unfortunately, we do not have any data on the methylation pattern, protein expression, and immunohistochemistry of SEMA3D due to resource limitations in our institution. Furthermore, our study utilized ARM patients' colon as controls. It has been shown that most ARM patients have abnormal colonic motility (41) and low expression of interstitial cells of Cajal marker (42). Therefore, further research with more proper control colon (e.g., autopsy bowel material from healthy infants or trauma patients) is necessary to better confirm the role of *SEMA3D* expression in the pathogenesis of HSCR. Noteworthy, however, our small sample size is a limitation of our study and suggests that a multicenter larger sample population needs to be studied to clarify our findings.

In conclusion, we report the first study of aberrant *SEMA3D* expressions in HSCR patients and suggest further understanding into the contribution of aberrant *SEMA3D* expression in the development of HSCR. In addition, this study is the first comprehensive analysis of *SEMA3D* variants in the Asian ancestry.

## Data Availability Statement

All data generated or analyzed during this study are included in the submission. The raw data are available from the corresponding author on reasonable request.

## Ethics Statement

The Institutional Review Board of the Faculty of Medicine, Public Health and Nursing, Universitas Gadjah Mada/Dr. Sardjito Hospital gave approval for this study (KE/FK/1356/EC/2015). All parents signed a written informed consent form before participating in this study.

## Author Contributions

G and KI conceived the study. G drafted the manuscript. KI and JV critically revised the manuscript for important intellectual content. AK, NB, and FR performed total RNA extraction and qPCR. HH, AM, MF, and DY conducted the experimental PCR-based work for Sanger sequencing. All authors have read and approved the manuscript and agreed to be accountable for all aspects of the work in ensuring that questions related to the accuracy or integrity of any part of the work are appropriately investigated and resolved.

### Conflict of Interest

The authors declare that the research was conducted in the absence of any commercial or financial relationships that could be construed as a potential conflict of interest.

## Supplementary Material

Supplemental Figure S1 Gel electrophoresis of qPCR: **(A)** *GAPDH* and **(B)** *SEMA3D* expressions for HSCR ganglionic, aganglionic, and control colons. M: 100 bp DNA ladder; G: ganglionic colon; A: aganglionic colon; C: control colon; W: no RNA.
