# Supplementary figures and images for: Aberrant Expressions and Variant Screening of SEMA3D in Indonesian Hirschsprung Patients
Source: Front Pediatr. 2020 Mar 11;8:60. doi: 10.3389/fped.2020.00060 (PMC7078240; doi:10.3389/fped.2020.00060)

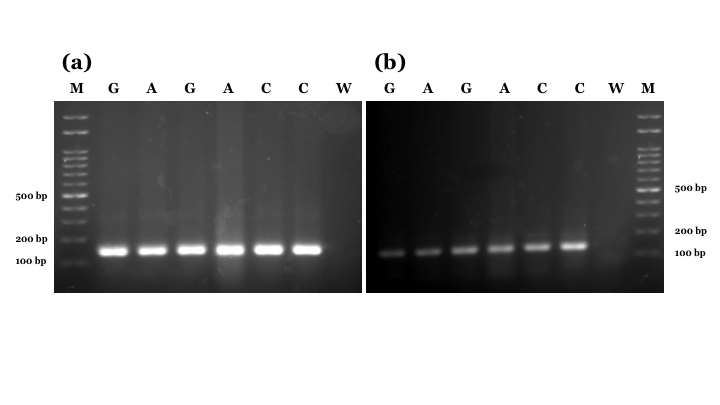

Supplement: Supplemental Figure S1 — Gel electrophoresis of qPCR: (A) GAPDH and (B) SEMA3D expressions for HSCR ganglionic, aganglionic, and control colons. M: 100 bp DNA ladder; G: ganglionic colon; A: aganglionic colon; C: control colon; W: no RNA. [file Image_1.TIFF]
